# Traumatic bone marrow lesions in dual-energy computed tomography

**DOI:** 10.1186/s13244-022-01312-6

**Published:** 2022-10-29

**Authors:** Qiuping Ren, Deqiu Tang, Zhiyuan Xiong, Heng Zhao, Shuixing Zhang

**Affiliations:** 1 Department of Radiology, The First Affiliated Hospital of Jinan University, No. 613, Huangpu West Road, Tianhe District, Guangzhou, 510627 Guangdong People’s Republic of China; 2 Department of Radiology, The First Affiliated Hospital, Hengyang Medical School, University of South China, Chuanshan Road No. 69, Hengyang, Hunan People’s Republic of China

**Keywords:** Trauma, Bone marrow lesions, Dual-energy computed tomography, Virtual non-calcium, Magnetic resonance imaging

## Abstract

Traumatic bone marrow lesions (TBMLs) are considered to represent a range of concealed bone injuries, including haemorrhage, infarction, and localised oedema caused by trabecular microfracture occurring in the cancellous bone. If TBMLs are not managed timeously, they potentially cause a series of complications that can lead to irreversible morbidity and prolonged recovery time. This article reviews interesting image findings of bone marrow lesions in dual-energy computed tomography (DECT). In addition to combining the benefits of traditional CT imaging, DECT also reveals and identifies various structures using diverse attenuation characteristics of different radiographic spectra. Therefore, DECT has the capacity to detect TBMLs, which have traditionally been diagnosed using MRI. Through evaluating DECT virtual non-calcium maps, the detection of TBMLs is rendered easier and more efficient in some acute accidents.

## Key Points

1. Traumatic bone marrow lesions represent a range of concealed bone injuries.
2. Dual-energy computed tomography and virtual non-calcium imaging have excellent diagnostic ability and proven utility for traumatic bone marrow lesion.
3. Radiologists can benefit from integrating dual-energy computed tomography and virtual non-calcium imaging into workflow.

## Introduction

Trauma induces a disruption of marrow trabeculae with leakage of interstitial fluid and haemorrhage to marrow spaces in Traumatic bone marrow lesion (TBML). TBML potentially causes various, severe clinical symptoms, leading to acute pain and joint-function loss, with or without other substantial soft-tissue injuries [1]. Without timely management, it may cause several complications, including dislocation, proteoglycan loss, chondrocyte degeneration, subchondral bone necrosis, and osteoarthritis, which potentially result in prolonged recovery time and irreversible morbidity [2]. In addition, after trauma, these reflect that the presence of TBML should lead to careful inspection and physical examination for other injuries changes to increase the diagnostic accuracy. Therefore, to prevent adverse patient outcomes, accurate and early diagnosis is prerequisite to optimal therapy.

MRI is the preferred imaging modality for diagnosing TBMLs. However, MRI has certain limitations in clinical practice, including pacemakers, claustrophobia, cochlear implants, and patient inability to remain stationary during image acquisition [3]. Conventional CT can detect fractures; however, TBMLs cannot be visualised due to the overlying trabecular bone [1]. By exploiting the photoelectric effect’s energy dependence in different radiographic spectra, dual-energy CT (DECT) facilitates the discrimination of different materials, decomposition of the three materials, and calculation of colour-coded virtual non-calcium (VNCa) images [4]. Therefore, dual-energy CT has enabled the detection of BMLs involving the absence of cortical disruption or with fractures [3–9].

In this review, we explore DECT’s emerging role in TBML imaging, with special focus on its diagnostic utility in delineating TBMLs [10].

## Definition of bone marrow lesion

BML is a common finding on MRI, describing a change in bone marrow signal strength on MRI, with or without low T1-weighted and enhanced signal intensity on fluid-sensitive sequences (T2/proton density with fat suppression and STIR) [11]. Notably, BML is not limited to bone injuries and can occur in infection, ischaemia, migrating osteoporosis, and early osteonecrosis and as a reaction to a tumour or as an idiopathic condition [11]. TBML is a post-traumatic bone marrow lesion, combined with bone marrow oedema, bleeding, cancellous bone trabecular microfracture, interstitial fluid exudation, and extracellular space bleeding, due to acute direct or indirect trauma (e.g. bone contusion), or by overload-induced subacute injury (e.g. stress fracture) [12]. Although TBMLs are often considered benign and self-limiting, they have recently been identified as a useful clinical phenomenon that may inform disease management [1], such as providing a potential target in TBML treatment that may precipitate osteoarthritis [10].

## Classification of bone marrow lesions

In 1988, Yao and Lee initially described irregular lesions on MRI in eight patients with acute knee injuries who had normal radiographs [13]. Berger et al. [14] described a series of 14 patients with radiographic occult fractures on MRI in the same year; nevertheless, these lesions were not classified. Bone bruising and occult fractures were separated a few years later [15]. Occult fractures are either radiographically undetectable or present minor abnormalities that are initially overlooked [16]. However, they exhibit MR characteristics remarkably similar to those of bone bruises, with the exception of an adjacent-cortex disturbance [15], whereas bone bruises or TBMLs exclusively involve the bone marrow [16].

Various classification systems have been used in different studies (Table 1). Lynch subsequently developed a three-type classification [17], based on morphology. Type I is described as diffuse inhomogeneous loss of reticular signal intensity on T1-weighted images within the medullary space of the cancellous bone and enhanced signal on sequences T2-weighted; type II lesions are identical to type I lesions; however, they have an interruption in the form of a smooth, black cortical line; type III lesions suggested as a considerable reduction of signal intensity, which was primarily limited to the immediate subcortical region on short TE images. However, intact or disrupted cartilage was not included in this classification. Bohndorf categorised the BMLs with intact (A) and disrupted (B) cartilage by integrating various findings at direct inspection (arthroscopy/arthrotomy) and the MR appearance of acute articular surface lesions, based on radiological and clinical findings [18]. Nevertheless, this classification combined clinical findings using arthroscopy/arthrotomy, which is an invasive event. Costa-Paz analysed BMLs using a three-level grading system, combined with both the appearance and location of BMLs [19]. Type 1: diffuse signal intensity, with change of medullar component; often reticular and distant from articular surface; type 2: localised signal intensity, with contiguity to articular surface; usually crescent in shape; type 3: disruption or depression of articular surface; often associated with type 2 lesions.

**Table 1:** Classification of bone marrow lesions

Classification system	Classification basis	Definition	
Lynch	Morphology	Type I is described as diffuse inhomogeneous loss of reticular signal intensity on T1-weighted images within the medullary space of the cancellous bone and enhanced signal on sequences T2-weighted	
		Type II lesions are identical to type I lesions; however, they have an interruption in the form of a smooth, black cortical line	
		Type III lesions suggested as a considerable reduction of signal intensity, which was primarily limited to the immediate subcortical region on short TE images	
Bohndorf	Radiological and clinical findings	Intact cartilage (type A)	Classic bone bruises: geographic and nonlinear subchondral area of low signal on T1-weigthed images and high intensity T2-weigthed images
			Subchondral impaction fractures: linear or pronged area of low signal on T1-weigthed images and high intensity T2-weigthed images that often extends vertically to the cortical bone and articular surfaces
		Disrupted cartilage (type B)	Chondral lesions and osteochondral lesions: simple cartilage fractures (“flake fractures”) with cartilage depression into the bone, osteochondral depression and partially or completely detached osteochondral flake fractures on MRI
Costa-Paz	The appearance and location of bone marrow lesions	Type 1 bone marrow lesions: diffuse signal intensity, with change of medullar component; often reticular and distant from articular surface	
		Type 2 bone marrow lesions: localised signal intensity, with contiguity to articular surface; usually crescent in shape	
		Type 3 bone marrow lesions: disruption or depression of articular surface; often associated with type 2 lesions	

Although several authors have attempted to improve these classification systems, Costa-Paz’s classification not only combined the appearance and location of BMLs but also evaluated both bone and cartilage non-invasively. The current studies focus on diagnostic performance of DECT for TBML. There are no studies about DECT for the TBML classification; however, its imaging appearance is similar with MR imaging finding. It is promising for classification of TBML in DECT. Therefore, the classification systems of TBML in MR are helpful to the understanding and diagnosis for DECT classification in TBML.

### Radiography

Most fractures can be diagnosed radiographically [20–22]. Computer X-ray and digital X-ray photography have improved the imaging quality significantly [23]. However, due to the projection-principle limitation, radiography has low-density resolution and cannot clearly reveal the overlapping and complex bone parts. Therefore, radiography often cannot diagnose TBMLs [24].

### Magnetic resonance imaging

TBMLs have widely been reported in the knee [10, 13, 15, 18, 25]. Yao and Lee describe these acute knee injuries as irregular signal on MRI, an unobvious or easily neglected phenomenon on plain radiographs [13]. MRI reveals BMLs as focal-signal abnormalities in the subchondral bone and bone marrow. A typical BML appears as a signal-loss area on T1 images and high signal intensity on T2 images owing to the wounded area’s increased fluid content. The optimal MRIs are produced from STIR sequences, where the signal from normal medullary fat is suppressed, thus highlighting BMLs with increased intensity. Although TBMLs have generally been diagnosed using MRI, which can identify characteristic findings, it necessitates prolonged acquisition times, and patients experiencing pain cannot remain in a fixed position, thus limiting MRI use in trauma settings.

### Positron emission tomography (PET)

In a prospective study, Marks et al. [26] suggested that bone scintigraphy can reveal bone injuries, which MRI confirmed as subchondral bone damage in 13 patients. Another study demonstrated that PET revealed several areas with increased tracer uptake that were colocalised by either bone or cartilage damage, thus providing additional information regarding bone marrow injuries [27]. Furthermore, additional morphologic imaging is recommended using ^18^F-Fluoride PET/MR because it can present BMLs more precisely and provide further diagnostic information at a higher diagnostic certainty [28]. Researchers have concluded that PET is a highly sensitive but moderately specific tool for detecting BMLs, with the drawback of low specificity. Therefore, PET is seldomly used for TBMLs.

### Ultrasonography (US)

US can sketch the cortical bone outline and adjacent soft-tissue changes at a fractured site using the high reflectivity at the cortical bone/peri-osseous soft-tissue interface [29]. Ultrasound is useful in bone and cartilage contour examinations when soft tissue surrounding the bone injury is painful and inflamed [30]. Moreover, when trauma history is obscure or a fracture is not suspected clinically, ultrasound can help diagnose occult fractures [31]. Additionally, US is a widely available, dynamic diagnostic imaging technology that is rapidly gaining traction as a cartilage evaluation tool [32]; however, most tibial- and patellar-cartilage parts in the knee joint are imperceptible [33]. Nevertheless, it is not the preferred TBML detection method [30].

### Single-energy computed tomography (SECT)

Standard SECT can accurately depict bone and even small fractures. SECT for occult fractures has highly sensitive and negative predictive values [34]. SECT of 0.625 mm axial-slice thickness, it is easy to assess patients with small or occult fractures with clinical suspicion of fracture and negative plain films, improving the time to diagnosis [35]. In particular, MDCT is a safe and appropriate first-line assessment tool for occult or small fractures [35]. Additionally, SECT yields substantial CT information and high resolution. It can perform three-dimensional reconstruction using surface shade display, volume rendering, maximum intensity projection, and other technologies [36]. Moreover, it reveals the fracture by any-plane reconstruction and is unaffected by overlapping shadows. It can clearly display fracture details and internal structure, which is an important means of diagnosing occult fracture. Therefore, SECT is an important and appropriate first-line investigation for early diagnosis of fracture, especially in occult or small fractures. However, SECT does not allow to evaluate bone marrow changes [6], it cannot facilitate TBML evaluation.

## DECT

### Principle of DECT

DECT’s ability to define and identify diverse body contents based on material degradation has recently increased due to technological advancements. DECT can distinguish different materials because high-density substances, such as iron, calcium, or iodine, exhibit variable attenuation at different X-ray spectrum energy levels at two separate energy levels, while conventional CT scan use a single polychromatic x-ray energy to acquire images with a single peak energy of around 100–120 kV [6, 37]. VNCa-image reconstruction can remove calcium from cancellous bone using a three-material decomposition model [4, 38]. The VNCa image, produced from the data of bone mineral composition removal (comprising calcium), can display the underlying bone marrow. VNCa-image colour coding allows qualitative assessment, while region-of-interest measurements provide quantitative assessment [39]. Consequently, DECT permits the characterisation and distinguishing of various body structures using varied attenuation at different X-ray spectra and allows increased visualisation of bone-marrow-oedema distribution for identifying BMLs. The specificity of DECT was highly dependent on reader experience and the acquisition technique. For reader experience, the specificity of DECT in the diagnosis of TBML was dependent on the experience of readers. Although inter-reader agreement was excellent for DECT-VNCa images, board-certified experienced readers had a higher specificity of 96% compared with trainees (residents or research students) [3]. A larger voltage difference (at least 60) can result in higher sensitivity between low and high kilovoltage for spectral separation [3]. In addition, single-source consecutive DECT showed lower specificity compared with the dual source CT; moreover, fast kilovoltage switching techniques showed similar estimates of diagnostic accuracy as a dual source technique compared with dual-layer DECT [3]. Different studies used different Rel.CM values for diagnosing TBMLs [5, 6, 8, 39, 40]; their results showed that the VNCa image with a Rel.CM value of 1.45 could be an optimal parameter. Therefore, for the evaluation of TBML on DECT in the clinical, we should try to read the images by certified experienced readers instead of trainees. For the sensitivity and specificity, we slightly recommend to use dual source and dual-layer DECT with a larger voltage difference (at least 60) and Rel.CM value of 1.45 can be an appropriate parameter for VNCa post-processing procedure.

### Application of DECT

DECT successfully detects TBMLs after acute trauma of the spine, knee, wrist, hip, and ankle in patients with trauma events using the VNCa technique, according to numerous previously published studies [4, 6, 8, 41–44]. Li’s meta-analysis evaluated the accuracy of DECT-VNCa imaging in detecting TBMLs and revealed DECT’s high sensitivity and specificity for detecting TBMLs; DECT-VNCa imaging performs considerably well in diagnosing TBMLs [45]. DECT is a potentially important future diagnostic modality for evaluating TBMLs.

#### Vertebral TBMLs

**Fig. 1:**
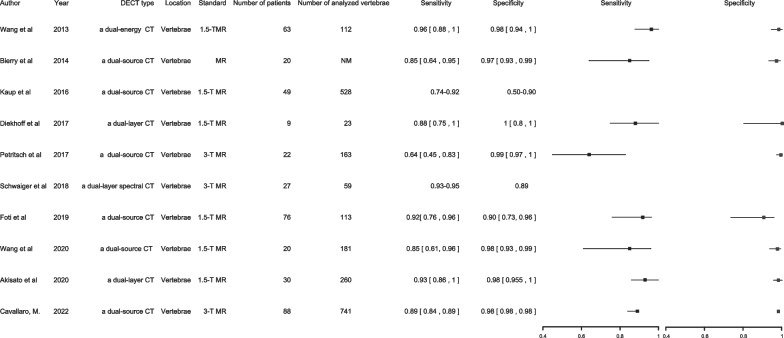
Vertebral TBMLs

DECT’s advancement has allowed it to directly assess BMLs present in acute fractures through VNCa-technique development, which is comparable to that of MRI [5, 38, 41, 43–49] (Fig. 1). This potentially enhances the diagnosis of patients with traumatic and osteoporotic fractures.

Yang et al. conducted a meta-analysis of seven studies involving 510 vertebrae, evaluated DECT’s accuracy in detecting TBMLs in patients suffering from vertebral-compression fractures used MRI as the reference standard, and found that the sensitivity, specificity, and AUC value of DECT for detecting TBML were 0.82, 0.98, and 0.965, respectively [41]. Ghazi analysed 17 studies involving 2468 vertebrae in 742 patients with BML on MRI and found DECT to have a high sensitivity, specificity, and AUC, pooled the sensitivity, specificity, and AUC of DECT for vertebral body TBML were 89%, 96%, and 96%, respectively [3]. These findings suggest that DECT offers impressive diagnostic accuracy for TBMLs in vertebrae, resulted a moderate sensitivity and a high specificity for TBML identification, and suggested that positive predictive value is higher than negative predictive value. In the diagnostic performance of quantitative assessment, some studies have some contradictions [38, 43, 44, 48]. Comparing with the qualitative evaluation, the quantitative evaluation has a lower specificity in DECT for TBMLs. Some studies supported that the quantitative evaluation is more dependent on the experience of the readers, complex post-processing technology, and individual patient differences [44, 50]. However, the high diagnostic performance of qualitative assessment is more important because it is more feasible to perform in in trauma settings, instead of quantitative measurement that requires additional time and effort. Since DECT’s high specificity and positive predictive value, the detection of TBML in DECT can help to increase radiologists’ confidence to diagnosis. It allows the detection of BMLs associated with vertebral fractures and may obviate emergency confirmatory MRI. Furthermore, DECT allows TBML detection, even in patients without evident fractures. Therefore, DECT is a promising diagnostic tool for detecting vertebral TBMLs.

#### Knee TBMLs

**Fig. 2:**
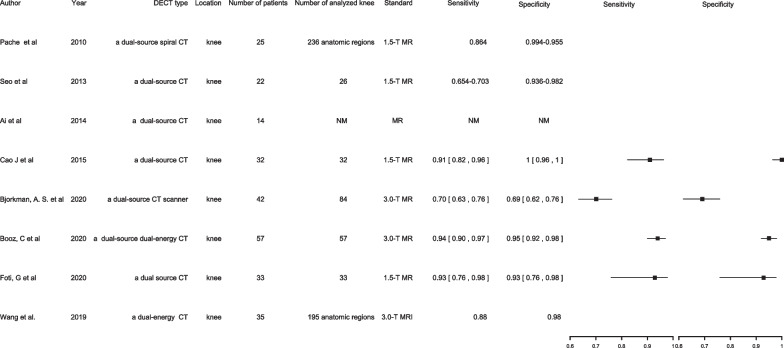
Knee TBMLs. *NM* Not mentioned

DECT exhibits similar diagnostic accuracy in depicting traumatic knee BMLs compared with vertebral TBMLs [8, 9, 39, 51–55] (Fig. 2). Previous TBML studies have concluded that DECT has high diagnostic accuracy, which is comparable to that of MRI.

According to a meta-analysis that used MRI as the reference standard, DECT demonstrates excellent specificity in evaluating TBMLs in patients with acute knee injuries [54]. However, DECT exhibits slightly lower sensitivity in detecting TBMLs than MRI, indicating that when DECT yields negative results in patients presenting symptoms, MRI may be required to further detect occult BMLs [54]. Moreover, several studies that have evaluated patients with acute knee injuries using DECT and MRI suggested that DECT is specific and accurate in detecting TBMLs in adult patients with acute knee injuries. DECT-VNCa reconstructions exhibit superb diagnostic performance in terms of sensitivity, specificity, and accuracy in depicting TBMLs compared with MRI [8, 39, 52, 53]. Furthermore, Booz’s study found DECT colour-coded map visualisation of TBMLs to have great diagnostic accuracy for TBML characterisation owing to its high-resolution and bone window compared with conventional CT images [39]. Moreover, in Foti’s study, DECT images improved the overall accuracy of TBML visualisation in chronic knee injuries [9]. These results confirmed DECT’s favourable diagnostic accuracy in depicting TBMLs around the knee joint both in acute and chronic traumatic situations compared with that of MRI. Ultimately, these results corroborate DECT’s excellent specificity and accuracy in detecting TBMLs in adult patients with suspected bone fractures or symptoms after acute knee injury, especially when MRI is contraindicated or unavailable.

#### Appendicular-skeleton (excluding knees) TBMLs

**Fig. 3:**
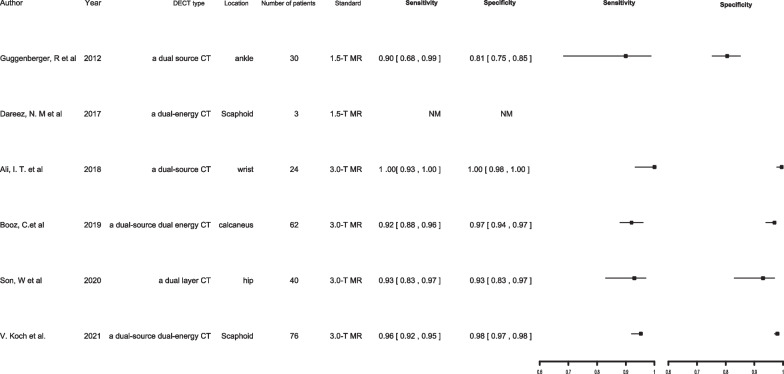
Appendicular-skeleton (excluding knees) TBMLs. *NM* Not mentioned

Recent studies have primarily investigated the DECT-VNCa technique’s diagnostic ability in detecting TBMLs in the appendicular skeleton, such as in the ankles, wrists, calcaneus, and lower limbs, compared with that of MRI [6, 42, 46, 56–58] (Fig. 3). They evaluated DECT’s diagnostic accuracy in detecting TBMLs throughout the appendicular skeleton. In a meta-analysis by Wilson, TBML detection in the appendicular skeleton by DECT was found to have excellent sensitivity, specificity, and AUC values [59]. According to Guggenberger’s study, DECT-reconstructed non-calcium images can help diagnose separate ankle-joint TBMLs with great sensitivity and negative predictive value and intermediate specificity and low positive predictive value [6]. Two studies demonstrated DECT’s utility in reliably identifying BMLs in the trauma wrist [42, 57]. DECT coupled with the VNCa algorithm was used to detect BMLs in wrist fractures and demonstrated reliability and user friendliness, rendering the DECT-VNCa algorithm a promising BML-detection technique in acute carpal trauma, with potential to improve CT’s diagnostic value in this injury type [42, 57]. Another study demonstrated the clinical applicability and high diagnostic accuracy of colour-coded DECT-VNCa reconstructions in the visualisation of calcaneus TBMLs compared with that of MRI [46]. This study demonstrated colour-coded-VNCa reconstruction’s comparability with MRI regarding diagnostic confidence and image noise, revealing no significant difference [46]. Accordingly, DECT exhibited high diagnostic accuracy and confidence in detecting TBMLs in patients with acute trauma injury to the appendicular system. Where MRI is unavailable or contraindicated, DECT can be performed as a feasible alternative for patients with acute trauma and probable BMLs, potentially leading to more efficient patient care after appendicular-skeleton trauma.

## Limitations and prospects of DECT for TBMLs

According to previous studies, there are certain limitations to DECT. First, DECT cannot provide enough information to assess soft-tissue injuries, such as those affecting periarticular ligaments, which are frequently injured in acute trauma accidents. Although DECT generally cannot assess soft-tissue injuries accurately, it is an alternative to MRI for patients with BMLs in appropriate circumstances and provides indirect signs that contribute to soft-tissue-injury assessment. Second, patients with negative DECT results and a high bone-abnormality suspicion may still require MRI to detect hidden bone marrow injuries. Third, DECT poses the risk of radiation exposure compared with MRI. Nevertheless, it is valuable in injury characterisation in acute trauma accidents; in addition to enabling the rapid delineation of fracture patterns, it can also detect BMLs without fractures and indirectly identify soft-tissue injuries.

Although MRI is a commonly used technique for BMLs, especially TBMLs, certain difficulties limit its usefulness for BML. MRI takes longer than CT; hence, patients who are in pain generally cannot maintain the same position for a long time. However, DECT has a short scanning time and no contraindications; moreover, it is applicable in emergency settings and involves individual clinical assistance. DECT enables perfect registration due to the almost simultaneous acquisition of different kVp data, which are not affected by breathing and movement artefacts [48]. Therefore, DECT is often the first option due to its increased availability, especially in emergency situations. DECT has shorter imaging duration and is helpful in patients who cannot assume a specific and rigid position. Additionally, TBMLs with small and subtle fractures cannot be discovered easily on MRI [60]. In addition to identifying fracture lines, DECT can also reveal TBMLs.

Consequently, DECT has developed the ability to visualise both BMLs and fracture lines, and it will serve as an alternative to MRI in future studies and applications, where MRI is unavailable or unfeasible. Despite certain limitations, when MRI is not available, DECT remains an excellent alternative to MRI, especially in acute trauma accidents. In TBML assessment, radiologists can benefit from integrating DECT and VNCa imaging into a radiology workflow for TBML portrayal and characterisation in trauma accidents and several acute trauma diagnostic challenges. Moreover, in acute trauma incidents, DECT is a potential ‘one-stop-shop’ evaluation tool, avoiding further patient repositioning and enhancing efficiency.

## Conclusion

DECT is a promising clinical application with potential benefits. DECT-VNCa imaging has excellent diagnostic ability and proven utility in TBML detection, and radiologists can improve the detection of TBMLs and TBMLs with subtle and occult fractures. DECT integration into TBML-assessment workflows in acute trauma accidents is set to increase, and DECT is a promising alternative to MRI.

## Data Availability

Not applicable.

## Abbreviations

BML: Bone marrow lesion
DECT: Dual-energy computed tomography
DECT: Dual-energy CT
PET: Positron emission tomography
SECT: Single-energy CT
STIR: Short-time inversion recovery
TBML: Traumatic bone marrow lesion
US: Ultrasonography
VNCa: Virtual non-calcium
